# Overcoming Obstacles to Drug Repositioning in Japan

**DOI:** 10.3389/fphar.2017.00729

**Published:** 2017-10-11

**Authors:** Yuhei Nishimura, Masaaki Tagawa, Hideki Ito, Kazuhiro Tsuruma, Hideaki Hara

**Affiliations:** ^1^Department of Integrative Pharmacology, Mie University Graduate School of Medicine, Tsu, Japan; ^2^Medical Affairs, Sumitomo Dainippon Pharma Co., Ltd., Tokyo, Japan; ^3^Department of CNS Research, Otsuka Pharmaceutical Co., Ltd., Tokushima, Japan; ^4^Molecular Pharmacology, Department of Biofunctional Evaluation, Gifu Pharmaceutical University, Gifu, Japan

**Keywords:** industry-sponsored clinical trial, investigator-initiated clinical trial, electronic health record, sharing resources, computational drug repositioning

## Abstract

Drug repositioning (DR) is the process of identifying new indications for existing drugs. DR usually focuses on drugs that have cleared phase-I safety trials but has yet to show efficacy for the intended indication. Therefore, DR can probably skip the preclinical and phase-I study, which can reduce the cost throughout drug development. However, the expensive phase-II/III trials are required to establish efficacy. The obstacles to DR include identification of new indications with a high success rate in clinical studies, obtaining funding for clinical studies, patent protection, and approval systems. To tackle these obstacles, various approaches have been applied to DR worldwide. In this perspective, we provide representative examples of DR and discuss the ongoing efforts to overcome obstacles to DR in Japan.

## Obstacles to DR in Japan

Drug repositioning (DR), also known as “drug repurposing,” seeks to develop new indications for existing drugs, to change the formulation, the dosage regimen, and the route of administration, and to create new combinations of drugs directed at multiple therapeutic targets. At present, the conventional *de novo* drug discovery process requires an average of about 14 years and US$2.5 billion to approve and launch a drug (Nosengo, 2016). The average time and costs to launch a drug *de novo* in Japan are 9.2 years and 55 billion yen, respectively (Yagi and Okubo, 2010). For a preclinical and phase-I study, it takes 1.3 and 1.1 billion yen per drug, respectively, in Japan (Yagi and Okubo, 2010). DR usually focuses on drugs that have cleared phase-I safety trials but has yet to show efficacy for the intended indication. Therefore, DR can probably skip the preclinical and phase-I study, thereby reducing the cost throughout drug development. Some estimates suggest that DR requires an average of ≈6.5 years and ≈US$300 million to approve and launch a drug (Naylor et al., 2015). However, the cost for a phase-III study remains expensive, and DR is not always a smooth and successful process (Nosengo, 2016). Pharmaceutical companies must have a clear path to economic return to justify the expense of a clinical trial for DR, considering the remaining patent life, new use patents, or data exclusivity (Shineman et al., 2014). This creates an opportunity that foundations and bioventures can help DR (Azvolinsky, 2017). Foundations can directly fund smaller proof-of-concept clinical trials for DR, and positive results from such trials may encourage further investment from government and/or industry to fund larger, multicenter trials. Bioventures can provide various pipelines for DR, such as *in silico* and/or *in vitro* screening platforms to identify DR candidates with a high success rate. Bioventures can also provide consultation on intellectual property to differentiate the DR candidates from those already marketed. DR can be used to obtain patent protection of a class of drugs in an unexpected indication as a method to block competitors from taking advantage of the new finding (e.g., pipeline management protection against competitors) (Mucke and Mucke, 2015). Currently, ≈10 patents related to DR are issued per month (Mucke, 2017). In the United States and Europe, there are many foundations and bioventures supporting DR, including Cure Within Reach (Bloom, 2015), Biovista (Deftereos et al., 2012), Insilico Medicine (Vanhaelen et al., 2017), and H.M. Pharma Consultancy (Mucke and Mucke, 2015). The regulatory frameworks in the US have also been changed to support DR. The US Food and Drug Administration Section 505(b)(2) permits the approval of applications for DR and permits reliance for such approvals on the information from previous studies about safety and/or effectiveness for an approved drug product. Applicants have the possibility to submit a 505(b)(2) application for a previously approved drug product (e.g., to support a new claim). These systems can significantly reduce the cost and effort of clinical trials and stimulate DR in various companies. In contrast, few bioventures support DR in Japan. The Ministry of Health, Labour, and Welfare (MHLW) and the Pharmaceuticals and Medical Devices Agency (PMDA), the regulatory agencies responsible for reviewing applications and approving the marketing authorization of drugs in Japan, do not have regulations equivalent to Section 505(b)(2). Much DR, however, has been successfully performed by both pharmaceutical companies and academia. Between 2001 and 2010, the PMDA gave 463 updated approvals, of which >60% were approvals for new indications of existing drugs (Hashitera et al., 2013). In this perspective, we provide representative examples of DR and the ongoing efforts to stimulate DR in Japan.

### Zonisamide

Zonisamide (1,2-benzisoxazole-3-methanesulfonamide) has been developed and marketed in Japan as an anti-epileptic drug by Sumitomo Dainippon Pharma (SDP) since 1989. Murata colleagues used zonisamide to treat epilepsy in a patient with Parkinson’s disease (PD) and serendipitously found that not only epilepsy but also symptoms related to PD were improved. They hypothesized that zonisamide could be repositioned for the treatment of PD and subsequently performed investigator-initiated clinical trials (IIT) in nine patients with PD (Murata et al., 2001). Seven patients showed a clear improvement in PD symptoms. Considering the positive finding, the patent life-cycle management, and the market potential, SDP decided to perform industry-sponsored clinical trials (IST) for the repositioning of zonisamide for PD. Phase IIb/III clinical trial were performed in 320 patients with PD, and patients treated with zonisamide again showed an improvement in PD symptoms. SDP then performed a phase III clinical trial on 133 patients with advanced PD, resulting in positive findings. Although the mechanism of action of zonisamide in both epilepsy and PD has not been fully elucidated, it may differ in each condition because of the 10-fold difference in approval therapeutic dose. The anti-epilepsy effect of zonisamide has been related to the inhibition of voltage-dependent sodium channels and T-type calcium channels (Holder and Wilfong, 2011). The anti-PD effect of zonisamide, however, may be related to the inhibition of dopamine metabolism via inhibition of monoamine oxidase-B (MAO-B), the stimulation of dopamine release from striatum, and the blockade of T-type calcium channels (Shahed and Jankovic, 2007; Sonsalla et al., 2010; Miwa et al., 2011). Based on these data, zonisamide was approved in Japan as an anti-PD drug under the brand name Trerief^®^ in 2009. The price of Trerief^®^ was determined by MHLW according to the price of selegiline, another anti-PD drug. This method of comparison based on similar efficacy means that the price of Trerief^®^ is significantly higher than that of Excegran^®^, the brand name of zonisamide as an anti-epileptic drug.

### Rebamipide

Rebamipide, a quinolinone derivative, has been developed and marketed in Japan for the treatment of peptic ulcer by Otsuka Pharmaceutical (OP) since 1990. The pharmacodynamics of rebamipide as anti-ulcer drug includes the stimulation of mucin secretion from goblet cells, resulting in the protection of gastric cells from various stimuli by coating the cell surface with a mucinous layer. Mucins are also expressed on the membranes of ocular surface epithelia and are secreted by conjunctival goblet cells, and thus play a role in ocular lubrication and defense. Reduced levels of mucins and changes in their distribution and glycosylation have been reported in patients with dry eye (Vickers and Gupta, 2015). However, therapies for dry eye that functioned by increasing mucin secretion in conjunctiva had not previously been developed. About 35% of the global population is affected by dry eye and this proportion is increasing, possibly as a result of lifestyle changes such as frequent computer and visual display usage (Vickers and Gupta, 2015). Considering the pharmacological effect of rebamipide on mucin secretion from goblet cells and the demanding need to develop novel therapies for dry eye, OP decided to reposition rebamipide for the treatment of dry eye. They demonstrated that rebamipide increased the production of mucin-like substances in the cornea and conjunctiva of a rabbit model of dry eye. They subsequently performed phase II and III ISTs and successfully demonstrated that a rebamipide ophthalmic suspension (unit dose 2%) improved both corneal and conjunctival epithelial damage and associated symptoms. Based on these studies, rebamipide was launched in Japan for the treatment of dry eye in 2012 under the brand name Mucosta^®^ ophthalmic suspension UD2%. The price of this formulation was set at 27 yen per 0.35 ml, based on the price of diquafosol sodium (Diquas^®^), which was developed by Santen as a *de novo* treatment for dry eye by increasing mucin secretion via activation of P2Y2 receptors in corneal epithelium (Nakamura et al., 2012). Diquas^®^ has been approved as a drug for dry eye since 2010 in Japan at a price of 641 yen per 5 ml, 1.6 times higher than that of Mucosta. A popular Japanese television program has promoted both Mucosta^®^ and Diquas^®^ as effective treatments for dry eye, and Mucosta^®^ has also been used to treat the condition in a well-known Japanese medical comedy show. These advertisements can have a significant impact on the public and effectively increase the sales of these drugs.

### Carperitide

Carperitide, a recombinant human atrial natriuretic peptide (ANP), has been used to prevent and/or treat cardiac failure. Nojiri et al. (2012) administered carperitide to patients undergoing lung cancer surgery to prevent post-surgical cardiovascular complications, especially in patients with cardiovascular disease risk factors. However, they serendipitously found that carperitide significantly reduced the metastasis of lung cancer after surgery. They studied the anti-cancer mechanism of carperitide using a mouse model of cancer metastasis and found that it inhibits cancer metastasis through suppression of E-selection in vascular endothelial cells, which decreases the attachment of cancer cells to vascular endothelial cells. They subsequently performed a retrospective study of 467 patients who underwent lung cancer surgery and confirmed that the survival ratio of patients treated with carperitide was significantly higher than that of patients without carperitide treatment. These studies strongly suggest that carperitide can be used to prevent lung cancer metastasis and improve the prognosis of patients with lung cancer (Nojiri et al., 2015). However, the patent for carperitide has expired and many generic ANP therapies are currently in clinical use, with the result that pharmaceutical companies have avoided investing in the repositioning of carperitide as an anti-cancer drug. After prolonged negotiations, Nojiri et al. (2017) have formulated a framework for an IIT that will be performed with the support of Shionogi, a pharmaceutical company based in Japan. The IIT, termed the JANP study, is currently recruiting patients.

### Thalidomide

Thalidomide was initially developed as a sedative and later repositioned to treat erythema nodosum leprosum and multiple myeloma because of its anti-angiogenic and anti-inflammatory effects. Kuwabara et al. (2008b) found that polyneuropathy, organomegaly, endocrinopathy, M-protein, and skin changes (POEMS) syndrome could be effectively treated with a combination of intensive chemotherapy and autologous peripheral blood cell-derived stem cell transplantation to suppress the proliferation of monoclonal plasma cells. These treatments, however, cannot be administered to patients with multiple organ failure and/or older age. Kuwabara et al. (2008a) hypothesized that thalidomide could be repositioned for the treatment of POEMS syndrome as it inhibits cytokine production and the proliferation of plasma cells. The authors had previously initiated an IIT in 2006 without pharmaceutical industry support and demonstrated a promising outcome in nine POEMS patients treated with thalidomide, later used as phase II data. Since 2010, Kuwabara et al. have performed a phase II/III IIT, the J-POST study, with the support of Fujimoto Pharmaceutical Corporation, the patent holder of thalidomide for multiple myeloma in Japan (Misawa et al., 2016). The IIT demonstrated that thalidomide reduced serum VEGF concentration and thus represents a novel treatment for patients with POEMS (Misawa et al., 2016). Approval for the use of thalidomide for this indication is pending from the PMDA.

## Future Directions

The examples of zonisamide and carperitide presented above demonstrate that serendipitous findings by astute clinicians are an important driver of DR and that the retrospective analysis of clinical records can be used to confirm the validity of these findings. Recent developments in electronic health records (EHRs) have made it possible to identify novel effects of clinical drugs in one EHR database and to validate these findings using other EHR databases (Xu et al., 2015). EHR can also be used to analyze similarity between diseases and between drugs without a prior knowledge of those diseases and drugs (Paik et al., 2015). For example, a novel relationship between disease A and B and drug X and Y can be identified using EHR. If a known relationship exists between disease A and drug X, one can hypothesize that there may also be a relationship between disease B and drug Y. Therefore, the sharing of EHR data between hospitals and of various clinical trial data in public databases can represent a powerful approach to computational DR. The PMDA is currently developing a public EHR database, Medical Information Database NETwork (MID-NET) (Noguchi, 2015), to be launched in 2018. Data from clinical trials can also be valuable resources for computational DR. Serious adverse events (SAEs) can be identified from randomized clinical trials. If a treatment arm has fewer predefined SAEs than the control arm, it is plausible that the drug used for the treatment arm is reducing the level of SAEs (Su and Sanger, 2017). Sharing data from clinical trials can increase the success rate of DR. The Study Data Tabulation Model (SDTM) from the Clinical Data Interchange Standards Consortium (CDISC) is a standard for creating a “data warehouse” for sharing the data from clinical research across studies. The PMDA has begun to request that the submission of data from clinical trials in accordance with the guidelines of CDISC (Ando, 2016). Tools that can generate SDTM data from clinical trials undertaken already without using CDISC guidelines have also been developed in Japan (Yamamoto et al., 2017). The PMDA and the Japan Agency of Medical Research and Development (AMED) are also developing the Clinical Innovation Network (CIN), a collaboration scheme with national medical research centers and industries, to share the clinical trials data between academia and industry (Hori, 2016). These frameworks may promote DR undertaken first by *in silico* analyses using big data, such as EHR and clinical-trial data, and then validated in IIT and/or IST.

High throughput screening of chemicals using *in vitro* and/or *in vivo* systems has also strongly driven DR (Nishimura and Hara, 2016; Imamura et al., 2017). The sharing of chemicals among researchers with various assay systems can increase the success rate of DR, as demonstrated by the success of projects supported by the National Center for Advancing Translational Sciences in the US (Azvolinsky, 2017). AMED has developed the Drug-Discovery Innovation and Screening Consortium, from which over 200,000 chemicals provided by 20 pharmaceutical companies are publicly available. Chemical libraries specialized in DR have also been provided by pharmaceutical companies within open innovation grants. These frameworks may promote DR firstly performed by *in vitro/vivo* screening, and then evaluated in an IST by the company holding the patent.

A machine-learning approach has also been popular in DR (Napolitano et al., 2013; March-Vila et al., 2017). Various algorithms of machine learning, including random forests (Cao and Moult, 2014), deep neural networks (Aliper et al., 2016), and deep adversarial networks (Kadurin et al., 2017), have been applied to DR combined with various omics databases, such as the Genome Wide Association Study Catalog (MacArthur et al., 2017) and the Library of Integrated Network-based Cellular Signature (LINCS) (Duan et al., 2014). These approaches have also been carried out successfully in Japan. For example, Iwata colleagues undertook an integrative approach using machine learning combined with multiple big data, including LINCS, Connectivity Map (Lamb et al., 2006), a Toxicogenomics Project-Genomics Assisted Toxicity Evaluation System (Igarashi et al., 2015), ChEMBL (Gaulton et al., 2017), Kyoto Encyclopedia of Genes and Genomes (Kotera et al., 2013), SuperTarget (Gunther et al., 2008), and DrugBank (Law et al., 2014) and found novel drug–protein–disease networks (Iwata et al., 2017). Asako and Uesawa (2017) developed a machine-learning model to predict agonists of the human estrogen receptor based on chemical structures.

The regulatory system for approval can affect the stream of DR significantly. A progressive approval system, in which the drug can be approved following proof of safety (Loike and Miller, 2017), may accelerate DR greatly, as suggested for rare diseases (Dunoyer, 2012) and regenerative medicine (Caplan and West, 2014). However, the risk of eliminating phase-II and -III studies should also be considered carefully (Loike and Miller, 2017). Conditional approval in Europe for orphan drugs (Dunoyer, 2012) and an approval system for off-label use of drugs validated in publicly funded research in Japan (Shimazawa and Ikeda, 2012) might serve as templates to consider the progressive approval system for DR. Lifecycle management, which includes patent protection and contributes to maximizing the return of investment for drug discovery, is another important aspect of DR. Analyses of lifecycle management in the Japanese market are undertaken actively (Hashitera et al., 2013; Yamanaka and Kano, 2016).

## Author Contributions

YN conceived and wrote the paper. MT, HI, KT, and HH wrote the paper.

## Conflict of Interest Statement

MT and HI have been employed by Sumitomo Dainippon Pharma and Otsuka Pharmaceutical, respectively. The other authors declare that the research was conducted in the absence of any commercial or financial relationships that could be construed as a potential conflict of interest.
